# Synthesis of Benzofuran Derivatives via Rearrangement and Their Inhibitory Activity on Acetylcholinesterase

**DOI:** 10.3390/molecules15128593

**Published:** 2010-11-29

**Authors:** Xiang Zhou, Miao Li, Xiao-Bing Wang, Tao Wang, Ling-Yi Kong

**Affiliations:** 1 Department of Natural Medicinal Chemistry, China Pharmaceutical University, 24 Tong Jia Xiang, Nanjing 210009, China; 2 Jiangsu Center for Drug Screening, China Pharmaceutical University, 24 Tong Jia Xiang, Nanjing 210009, China

**Keywords:** coumarin, benzofuran, rearrangement, anti-AChE

## Abstract

During a synthesis of coumarins to obtain new candidates for treating Alzheimer’s Disease (AD), an unusual rearrangement of a benzopyran group to a benzofuran group occurred, offering a novel synthesis pathway of these benzofuran derivatives. The possible mechanism of the novel rearrangement was also discussed. All of the benzofuran derivatives have weak anti-AChE activities compared with the reference compound, donepezil.

## 1. Introduction

Benzofuran derivatives are a major group of biologically active heterocycles, which are usually important constituents of plant extracts used in medicinal chemistry for their various biological activities [1,2,3,4]. Due to their diverse activities, much attention has been paid to synthetic strategies to access these systems, and a number of methods have been developed [5,6,7], but a method involving the rearrangement from a benzopyran group to a benzofuran group has not been reported before. More interesting to us, studies have shown that some of benzofuran derivatives such as donepezil [8] were able to inhibit acetylcholinesterase (AChE), or had the capability of reducing aggregated beta-amyloid (Aβ) in the brain [9,10]. This prompted us to continued synthesizing benzofuran derivatives through the rearrangement of coumarins to expand our efforts on developing novel AChE inhibitors for treating Alzheimer’s disease (AD).

**Figure 1:**
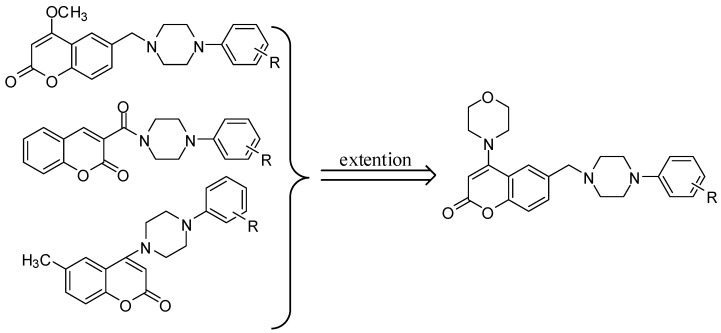
Design strategy of the target compounds.

As we know, AChE and toxic Aβ are the main medication targets for treating AD so far, so dual binding AChE inhibitors [11], which can not only facilitate cholinergic transmission but also interfere with AD pathogenesis [12,13], namely the synthesis, deposition and aggregation of toxic Aβ in brain areas, have become the leading strategy for the development of anti-AD agents [14,15,16,17]. Hence developing novel dual binding AChE inhibitors is our particular interest and as part of our research program on naturally occurring biological coumarins [18,19,20], a series of novel coumarin derivatives has already been designed and synthesized aiming at AChE inhibitory activity [21]. As the extension of our efforts on developing new AChE inhibitors based on the coumarins, a novel series of derivatives were designed (Figure 1), but surprisingly, we did not obtain the target compounds during synthesis but rather an unusual rearrangement of the benzopyran group to a benzofuran group occurred instead, which offered a new synthetic pathway of benzofuran derivatives not reported before. Here, the details of the new rearrangement as well as the pharmacological characteristics of the new benzofuran derivatives obtained through this rearrangement are discussed.

## 2. Results and Discussion

### 2.1. Chemistry

**Scheme 1:**
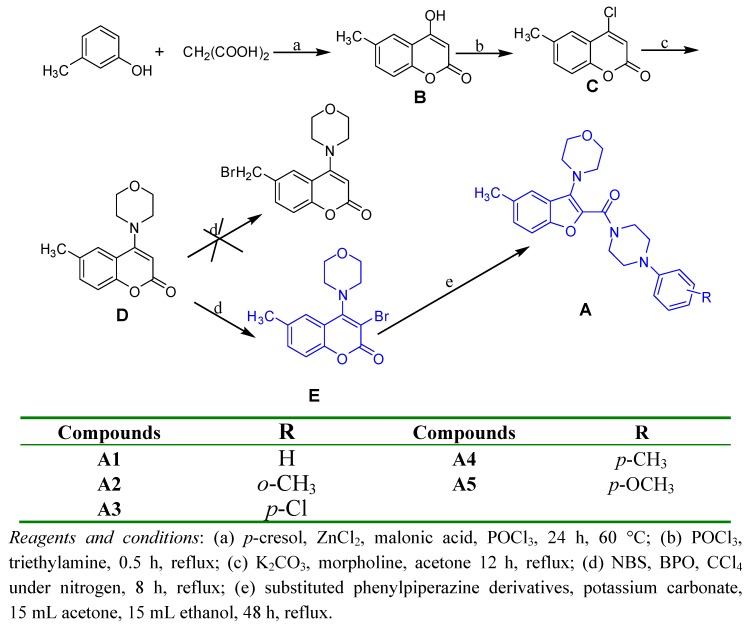
The preparation of the target compounds.

The synthesis of the benzofuran derivatives is shown in **Scheme 1**. The key intermediate 4-hydroxy-6-methylcoumarin (**B**) was prepared from *p*-cresol and malonic acid using ZnCl_2_ as the catalyst in POCl_3_ and the mixture was stirred at 60 °C for 24 h to give **B**. Then **B** was treated with POCl_3_ at reflux for 0.5 h to give 4-chloro-6-methylcoumarin (**C**). 4-morpholino-6-methylcoumarin (**D**) was obtained through the reaction between **C** and morpholine under reflux for 12 h. Compound **D** was brominated with NBS using BPO as the initiator to give 3-bromo-6-methyl-4-morpholinocoumarin (**E**) and not the expected 6-(bromomethyl)-4-morpholinocoumarin. Finally, **E** reacted with the corresponding piperazine substituents [22] and the rearranged compounds **A1-A5** were obtained.

**Figure 2:**
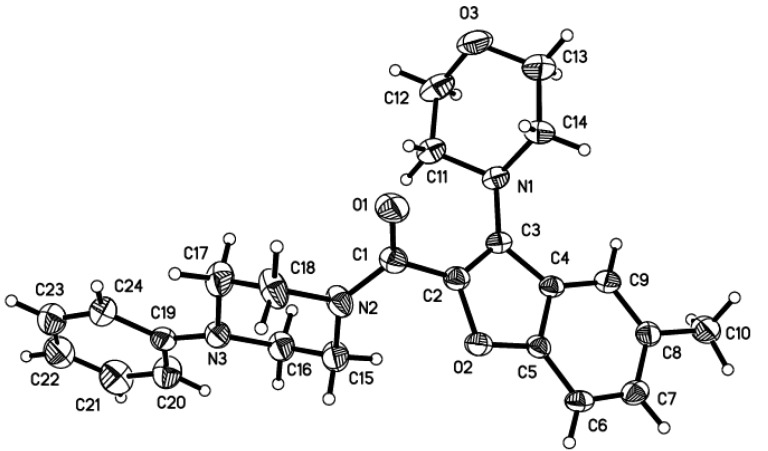
The X-ray diffraction structure of compound **A1**.

The structures of the target compounds were elucidated by ^1^H-NMR, IR and ESI-MS. In order to confirm their chemical structures, one of the target compounds, **A1**, was further identified by single crystal X-ray diffraction (Figure 2).

**Scheme 2:**
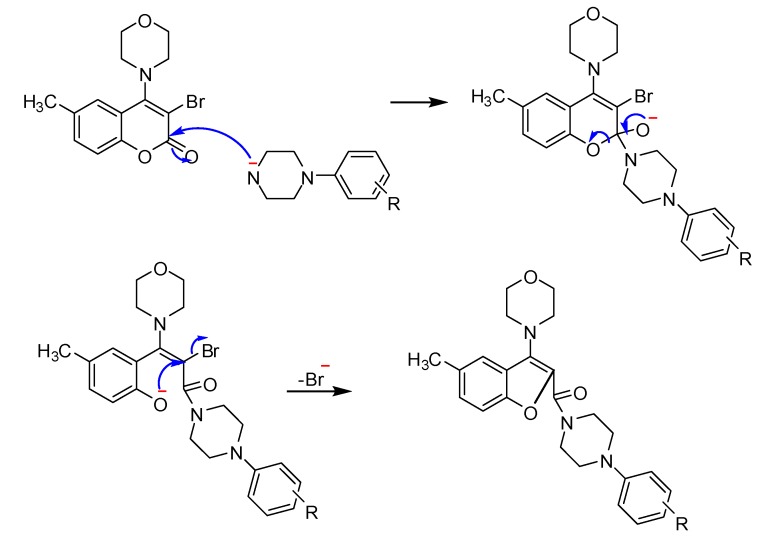
A possible mechanism of the rearrangement.

Through analyzing its chemical spectral data, the structure of **D** was identified as 6-methyl-4- morpholinocoumarin and that of **E** as 3-bromo-6-methyl-4- morpholinocoumarin and not the expected 6-(bromomethyl)-4-morpholinocoumarin, but **A1** was identified as (4-phenylpiperazin-1-yl) (5-methyl-3-morpholino-benzofuran-2-yl) methanone, so it can be concluded that the rearrangement should have occured in the step of synthesizing **A** from **E**. A possible mechanism of this transformation (shown in Scheme 2) is also discussed below. As to the position of bromination, after comparing the structure of **D** with the coumarin derivatives synthesized before [21], it can be noticed that the main difference between them was the substituents on the 4-position of the coumarin group, which was a basic morpholino group in **D**, while in the coumarin derivatives synthesized before it was a methyl, methoxy or chloro group, so the substituents on the 4-position of the coumarin group should be taken into account during the study of the position of bromination..

### 2.2. Anti-AChE testing

To determine the therapeutic potency of the benzofuran derivatives **A1-A5** for treating AD, their anti-AChE activities were assayed according to Ellmann’s method [23], utilizing freshly prepared AChE from rats brain homogenate and donepezil as the reference compound. Inhibition of AChE activities of them was shown in Table 1.

**Table 1:** Inhibition of AChE activities of the synthesized compounds.

**Compound**	AChE inhibition (IC50, μmol/L) ^a^	Compound	AChE inhibition (IC50, μmol/L) ^a^
**A1**	45 ± 0. 3	**A4**	11 ± 0.2
**A2**	32 ± 0.1	**A5**	21 ± 0.1
**A3**	73 ± 0. 2	donepezil	0.11 ± 0.01

^a^ Data are means ± standard deviation of three independent experiments.

In general, the data in **Table 1** clearly shows that all of these compounds exhibited moderate inhibition activities toward the cholinesterase. Among them compound **A4** (4-(p-tolylpiperazin-1-yl) (5-methyl-3-morpholinobenzofuran-2-yl) methanone showed the best AChE inhibitory activity with the IC_50_ value of 11 μmol/L.

## 3. Conclusions

By linking a substituted phenylpiperazine moiety to the benzofuran backbone conformationally restricted derivatives that are as potential inhibitors of AChE for treating AD were synthesized. An unusual rearrangement from a benzopyran group to a benzofuran group occurred. The rearrangement ocurred under moderate conditions and offers a facile and practical preparation of biologically active benzofuran derivatives, so it may be worthwhile for developing potential AChE inhibitors through the rearrangement of benzopyran rings to benzofuran derivatives under mild conditions.

## 4. Experimental

### 4.1. General

Reaction progress was monitored using thin layer chromatography (TLC) on precoated Merck silica gel Kiesegel 60 F254 plates and the spots were detected under UV light (254 nm). Flash chromatography was performed with 230–400 mesh silica gel. The IR spectra were measured on a Jasco FT/IR-430 spectrophotometer. Melting points (mp) were obtained on a B-540 Buchi melting-point apparatus and are uncorrected. The ^1^H-NMR spectra were recorded on a 500 MHz Bruker spectrometer. The chemical shifts are reported downfield in ppm relative to internal TMS, and coupling constants are reported in Hertz (Hz). Mass spectra were run on a HP 5989A electrospray ionization mass spectrometer spectrometer. The mass spectra analysis is reported as m/z values.

### 4.2. 4-Chloro-6-methylcoumarin *(**C**)*

Compound **B** (0.005 mol) and triethylamine (0.005 mol) were added to POCl_3_ (5 mL). The mixture was reﬂuxed for 30 min, and poured into water. Then the solution was extracted with methylene chloride. The methylene chloride layer was dried with anhydrous Na_2_SO_4_ and evaporated under reduced pressure. Flash chromatography (cyclohexane-acetone = 10:1) was performed to give **C** 0.8 g (yield 82.3%); ^1^H-NMR (CDCl_3_) δ: 7.56 (d, 1H, H-5, *J* = 2.1 Hz), 7.26 (dd, 1H, H-7, *J* = 2.1 Hz, 8.7 Hz), 7.28 (d, 1H, H-8, *J* = 8.7 Hz), 6.53 (s, 1H, H-3), 2.39 (s, 3H, 6-CH_3_).

### 4.3. 6-Methyl-4-morpholinocoumarin *(**D**)*

**C** (0.01 mol), K_2_CO_3_ (0.02 mol) and morpholine (0.01 mol) were dissolved in acetone (50 mL), then the mixture was refluxed for 12 h., after which the reaction mixture was filtered and the filtrate was evaporated under reduced pressure. Flash chromatography (cyclohexane-acetone = 10:1) was performed to give **D** 0.97 g (yield 75.6%); ^1^H-NMR (CDCl_3_) δ: 7.36 (d, 1H, H-5, *J* = 1.9 Hz), 7.31 (dd, 1H, H-7, *J* = 1.9 Hz, 8.5 Hz), 7.24 (d, 1H, H-8, *J* = 8.5 Hz), 5.73 (s, 1H, H-3), 3.24 [m, 4H, N_1_(CH_2_)_2_], 3.94 [m, 4H, (CH_2_)_2_O], 2.41 (s, 3H, 6-CH_3_).

### 4.4. 3-Bromo-6-methyl-4-morpholinocoumarin *(**E**)*

**D** (0.003 mol) were added to anhydrous CCl_4_ (34 mL) with the reaction system protected by N_2_. The mixture was refluxed for 0.5 h, then 1-bromopyrrolidine- 2, 5- dione (NBS, 0.0028 mol) and methyl benzoperoxoate (BPO, 100 mg) were added to the reaction mixture. After the reaction refluxed for 5 h, additional BPO (100 mg) was added again, and the reaction continued for 3 h. Then the reaction mixture was cooled down to room temperature and filtered. The filtrate was evaporated under reduced pressure. Flash chromatography (cyclohexane-acetone = 5:1) was performed to give **E** 0.32 g (yield 59.6%); ^1^H-NMR (CDCl_3_) δ: 7.36 (m, 1H, H-9), 7.34 (dd, 1H, H-7, *J* = 1.9 Hz, 8.4 Hz), 7.24 (d, 1H, H - 6, *J* = 8.4 Hz), 3.54 [m, 4H, N_1_(CH_2_)_2_], 3.93 [m, 4H, (CH_2_)_2_O], 2.44 (s, 3H, 10-CH_3_). ^13^C-NMR (CDCl_3_): 169.3 (C-3); 157.2 (C-1); 149.8 (C-8); 136.2 (C-5); 132.3 (C-6); 127.7 (C-4); 117.3 (C-7); 114.9 (C-9); 75.8 (C-2); 67.7(C-11 and C-12); 48.9 (C-10 and C-13); 22.3 (CH_3_). ESI-MS: 325.3 ([*M*+H]^+^).

### 4.5. General synthetic procedure for target compounds ***A***

**E** (1 mmol) was added to a mixture of acetone (15 mL) and ethanol (15 mL) containing a substituted piperazine derivative (1 mmol) and potassium carbonate (2 mmol). The mixture was refluxed for 48 h and the reaction mixture was evaporated. Flash chromatography was performed to afford the target compounds that were further purified by recrystallization from ethanol.

*(5-Methyl-3-morpholinobenzofuran-2-yl)-(4-phenylpiperazin-1-yl) methanone* (**A1**) Yield 49%; colorless powder; mp 131–133 °C; IR (KBr) ν cm^−1^: 3449, 2826, 1620, 1598, 1464, 1338, 1260, 1200, 1021, 902. ^1^H-NMR (CDCl_3_) δ: 7.58 (s, 1H, H-9), 7.44 (m, 1H, H-7), 7.22~7.28 (m, 3H, Ph, H - 6), 6.82~7.00 (m, 3H, Ph), 3.61~3.84 (m, 8H, CH_2_-15, CH_2_-18, O(CH_2_)_2_), 3.15~3.20 (m, 8H, CH_2_-11, CH_2_-14, CH_2_-16, CH_2_-17), 2.42 (s, 3H, CH_3_-10); ESI-MS: 406.2 ([*M*+H]^+^). CCDC number 740474 contains the supplementary crystallographic data for this compound. These data can be obtained free of charge from The Cambridge Crystallographic Data Centre via www.ccdc.cam.ac.uk/data_request/cif. Empirical formula, C_24_H_27_N_3_O_3_; molecular weight, 405.49; crystal dimensions, 0.40 × 0.34 × 0.25 mm; Orthorhombic, Pbca; a = 15.385 (2) Å, b = 7.301 (2) Å, c = 37.579 (3) Å; α = 90.00°, β = 90.00°, γ = 90.00°; V = 4221.3 (14) Å3; Z = 8; Dx = 1.276 Mg· m-3; R1 = 0.0546; ωR2 = 0.1205; GOOF = 1.055; θ = 1.71-25.01°; μ = 0.085 mm-1; T = 298(2)K.

*(4-o-Tolylpiperazin-1-yl)-(5-methyl-3-morpholinobenzofuran-2-yl) methanone* (**A2**) Yield 43%; colorless powder; mp 122–125 °C; IR (KBr) ν cm^−1^: 3424, 2954, 1617, 1518, 1385, 1259, 1158, 802; ^1^H-NMR (CDCl_3_) δ: 7.42 (s, 1H, H-9), 7.37 (m, 1H, H-7), 7.17 (m, 1H, H-6), 7.08~7.10 (m, 2H, Ph), 6.85~6.87 (m, 2H, Ph), 3.86~3.95 [m, 8H, CH_2_-15, CH_2_-18, O(CH_2_)_2_], 3.19~3.29 (m, 8H, CH_2_-11, CH_2_-14, CH_2_-16, CH_2_-17), 2.42 (s, 3H, CH_3_-10), 2.42 (s, 3H, CH_3_). ESI-MS: 420.2 ([*M*+H]^+^).

*(4-(4-Chlorophenyl)piperazin-1-yl)-(5-methyl-3-morpholinobenzofuran-2-yl) methanone* (**A3**) Yield 42%; colorless powder; mp 128–130 °C; IR (KBr) ν cm^−1^: 2955, 2862, 1620, 1593, 1499, 1387, 1259, 1231, 813. ^1^H-NMR (CDCl_3_) δ: 7.43 (s, 1H, H-9), 7.29 (m, 1H, H-7), 7.19~7.24 (m, 3H, Ph, H-6), 6.85~6.87 (m, 2H, Ph), 3.73~3.88 [m, 8H, CH_2_-15, CH_2_-18, O(CH_2_)_2_], 3.21~3.29 (m, 8H, CH_2_-11, CH_2_-14, CH_2_-16, CH_2_-17), 2.42 (m, 3H, CH_3_). ESI-MS: 440.1 ([*M*+H]^+^).

*(4-p-Tolylpiperazin-1-yl)-(5-methyl-3-morpholinobenzofuran-2-yl) methanone* (**A4**) Yield 43%; colorless powder; mp 122–125 °C; IR (KBr) ν cm^−1^: 2968, 2832, 1623, 1597, 1494, 1270, 1200, 1024, 917, 768; ^1^H-NMR (CDCl_3_) δ: 7.42 (s, 1H, H-9), 7.30 (m, 1H, H-7), 7.16~7.21 (m, 3H, Ph, H-6), 7.01~7.04 (m, 2H, Ph), 3.70~3.90 [m, 8H, CH_2_-15, CH_2_-18, O(CH_2_)_2_], 2.96~3.31 (m, 8H, CH_2_-11, CH_2_-14, CH_2_-16, CH_2_-17), 2.45 (s, 3H, CH_3_-10), 2.34 (s, 3H, CH_3_). ESI-MS: 420.2 ([*M*+H]^+^).

*(4-(4-Methoxyphenyl)piperazin-1-yl) (5-methyl-3-morpholinobenzofuran-2-yl) methanone* (**A5**) Yield 39%; colorless powder; mp 161–163 °C; IR (KBr) ν cm^−1^: 2914, 2828, 1619, 1514, 1461, 1228, 1157, 816. ^1^H-NMR (CDCl_3_) δ: 7.42 (s, 1H, H-9), 7.29 (m, 1H, H-7), 7.17 (m, 1H, H-6), 6.91 (m, 2H, Ph), 6.85~6.87 (m, 2H, Ph), 3.86~3.95 [m, 8H, CH_2_- 15, CH_2_-18, O(CH_2_)_2_], 3.12~3.29 (m, 8H, CH_2_-11, CH_2_-14, CH_2_-16, CH_2_-17), 3.77 (s, 3H, OCH_3_), 2.42 (m, 3H, CH_3_-10). ESI-MS: 436.2 ([*M*+H]^+^).

### 4.6. *In vitro* AChE inhibition assay

AChE activity was measured in duplicate by the spectrophotometric method reported by Ellman *et al.* [23] with some modifications, mainly involving the source of the enzyme and the reference compound. Rat brain homogenate was used as the enzyme source. The whole brain except for the cerebellum was homogenized in nine volumes of 100 mM sodium phosphate buffer (pH 7.0). The test compounds were dissolved in dimethyl sulphoxide (DMSO). The AChE activity was expressed as a change in OD at 412 nm.
